# Assessment of musculoskeletal symptoms and their impacts in the adolescent population: adaptation and validation of a questionnaire

**DOI:** 10.1186/1471-2431-14-173

**Published:** 2014-07-03

**Authors:** Élise P Legault, Vincent Cantin, Martin Descarreaux

**Affiliations:** 1 Département des sciences de l’activité physique, Université du Québec à Trois-Rivières, 3351 boul. des Forges, C.P. 500, Trois-Rivières, Québec G9A 5H7, Canada

**Keywords:** Musculoskeletal symptoms, Adolescents, Musculoskeletal disorders, Validation

## Abstract

**Background:**

Valid and reliable instruments measuring musculoskeletal symptoms prevalence and their impacts in the adolescent population are scarce. The Extended Nordic Musculoskeletal Questionnaire (NMQ-E) is a reliable instrument that measures the prevalence, severity and impact of musculoskeletal symptoms. The purpose of this study was: (1) to develop a musculoskeletal symptom screening tool for younger populations derived from the NMQ-E and NMQ French versions and (2) to assess the validity and reliability of the adapted version of the instrument.

**Methods:**

Based on the results of a translated (French) and adapted NMQ-E administered to 61 adolescents, a final 27-item dichotomous questionnaire was developed. The questionnaire measured the 6-month prevalence of musculoskeletal symptoms and the impact of these symptoms on school attendance as well as on sports and leisure activity participation. Among the adolescents who agreed to participate, thirty-nine (mean age: 13.7 ± 1.8) formed the reliability cohort and thirty-four (mean age: 14.2 ± 2.3) formed the criterion validity cohort. Reliability was measured by test-retest with a mean time interval of 28 hours. Criterion validity was assessed by comparing the answers to the questionnaires to the participants’ clinical records. Statistical tests used were proportions of observed agreement (Po) and the Cohen kappa statistic (k).

**Results:**

The mean Po for the test-retest was 0.92 for the 6-month symptom prevalence items, 0.99 for the impact of symptoms on school items and 0.96 for the impact on sports and leisure activities items. Kappa values for the reliability assessment ranged between 0.57 and 1.00 for the 27 dichotomous variables. The criterion validity kappa obtained for the agreement between participants’ clinical records and questionnaires was k = 0.76.

**Conclusions:**

Kappa values for the reliability and the criterion validity are of moderate to perfect agreement beyond chance, indicating that there are only minor variations between tests, and good agreement between questionnaire items and clinical records. These results indicate that the adapted version of the NMQ-E is an appropriate self-administered musculoskeletal symptom screening tool for the adolescent population. Items related to the impacts of symptoms would benefit from additional validation using school and sport attendance records.

## Background

Musculoskeletal symptoms are prevalent in the adolescent population and often have significant impact on their future musculoskeletal health. One study identified musculoskeletal pain as the second most reported physical symptom after headaches, and up to 7% of adolescents report this type of symptom often or on a daily basis [1]. In Ontario (Canada) alone, 380 000 adolescents and pre-adolescents consulted a health provider for musculoskeletal disorders over the course of a year, which represents a consultation rate of 122 visits per 1000 youths [2]. Low back pain, more precisely, is one of the most prevalent complaints in the adolescent population. Studies found annual prevalence of low back pain varying between 20.5 and 50% during adolescence [3-6]. Adolescents who have low back pain are also more likely to develop chronic low back pain as adults [7-9]. The development of musculoskeletal disorders affecting the spine at a younger age is to be taken seriously considering the potential risk of developing chronic low back pain.

In the active adolescent population, sport and recreational injuries are also common. According to a United-States National medical care survey conducted over a year, the number of emergency department visits for the treatment of injuries due to physical activity and sports was estimated at 2.6 million for people aged between 5 and 24 years [10]. In Canada, 27% of adolescents aged between 12 and 19 years old suffered from at least one injury in 2009, and 66% of these injuries occurred during physical or sporting activities [11]. Sports injuries in the adolescent population are on the rise with the proportion of injuries due to sport and physical activity having increased by 5% between 2001 and 2009 [11].

The survey of musculoskeletal symptoms in the adolescent population, be they related to a sport injury or not, is an important component in the detection and the prevention of musculoskeletal injury or pain and their related consequences. Furthermore, this type of screening tool is often used in the development and assessment of prevention strategies for work related pain and symptoms. [12,13]. Surveillance programs similar to those seen in work safety and ergonomics could be useful for school physical activity programs or for individual and team sports.

Epidemiological research on symptoms or injuries can be conducted using clinical records, nationwide surveys or questionnaires. Some of these methods, however, can underestimate the prevalence and incidence of symptoms. Surveillance through hospital records rarely accounts for symptoms or injuries that are treated by other health practitioners (chiropractors, physiotherapists, etc.), nor does it account for the minor disorders that remain untreated [13-15]. Retrospective questionnaires are another method of estimating the actual prevalence of musculoskeletal symptoms, which offer a smaller risk of underestimating or even leaving out minor symptoms. Questionnaires are also a good method to obtain information from a large, and therefore more representative, population sample. Various questionnaires and assessment tools have been shown to be valid [16,17], reliable [17,18] and cost-efficient [19] when collecting injury data in the youth population. However, to our knowledge, questionnaires measuring musculoskeletal symptoms that are also adapted to the adolescent population are not currently available.

In order for retrospective questionnaires to be valid and reliable, the recall period must be reasonably short, as recall bias limit data validity depending on the level of detail requested and the severity of the injuries [20,21]. *Harel et al*. [21] assessed the recall capacities of parents when reporting their children or adolescents injuries over a period varying between 2 weeks to 12 months. The authors of this study concluded that severe injuries resulting in either hospitalisation or one full school day loss are less likely to be affected by recall bias due to memory decay [21]. On the other hand, minor injuries are affected by memory decay, especially if the recall period exceeds 5 months [21]. These studies, however, refer to injuries rather than symptoms, and were conducted in an adult population. It is to be noted that recall bias may be slightly different in adolescent populations and when musculoskeletal symptoms are assessed, rather than injuries.

Despite the numerous studies using questionnaires to collect epidemiological information, few validated musculoskeletal symptoms survey instruments exist. One of the commonly used tools is the Nordic Musculoskeletal Questionnaire (NMQ), a validated instrument that was originally developed to study the prevalence and impact of work related musculoskeletal symptoms [22]. The NMQ, in its extended version (NMQ-E), measures the point, 12-month and lifetime prevalence of musculoskeletal symptoms [23]. The NMQ-E also measures the severity of the symptoms by assessing the impact of the disorder on work and leisure activities. Questions on the treatment of the disorder such as hospitalisation are also used to estimate symptom severity [13]. Finally, the NMQ-E is an easy to use, one-page questionnaire designed to obtain wide-ranging information on musculoskeletal symptoms over nine body regions in a short time frame. This questionnaire has, however, never been adapted to younger populations. An adapted version of this questionnaire would be an easy-to-use tool to survey symptom prevalence and severity, thus making it easier to identify and prevent musculoskeletal problems in the adolescent population.

Given the lack of validated musculoskeletal symptom survey instruments, the first objective of this study was to develop a musculoskeletal symptom screening tool for younger populations derived from the NMQ-E and NMQ French versions. The second objective of this study was to determine both the reliability and validity of this adapted version of the NMQ-E.

## Methods

The original and the extended versions of the NMQ questionnaire assess the lifetime, 12-month and point prevalence of musculoskeletal symptoms of nine body regions. The severity of the symptoms is also assessed by this instrument. Its extended (NMQ-E) version has a convenient one-page design that contains 99 questions and that can be completed in approximately 10 to 15 minutes. This latest version, along with other translated French versions of the NMQ [13,24], were used to develop the questionnaire of the present study.

### Face validity

Face validity is an interpretive and subjective measure of an instrument by its users [25]. It is the first step in the validity process and is used to determine if the questionnaire is easily and correctly understood by its users. The face validity of the first questionnaire draft was initially assessed amongst 61 adolescents, aged between 11 and 16 years (mean: 13.3 ± 1.1). Participants were instructed to complete the questionnaire individually. Two researchers were present during the face validity trials in order to write down the participants’ questions and comments, as well as the time taken to complete the questionnaire. The adolescents were also instructed to highlight any questions or words that they had difficulty understanding. Once the questionnaires were completed, five adolescents participated in a brief focus group session during which their interpretation of the instrument was discussed. The proportion of correctly answered questionnaires was also calculated. The conditions that had to be satisfied for the questionnaire to be considered successfully completed were: 1) no incoherently answered questions, and 2) no missing answers. Results of the face validity analysis are detailed in the results section.

### Questionnaire description – final version

The resulting questionnaire, following the face validity analysis, contained a total of 27 questions: three questions per body region in a three-page format. The life prevalence of musculoskeletal symptom and its related questions were removed due to the high probability of recall bias. Rather, a 6-month recall period was chosen for the prevalence questions on the final version. Previous studies show that a longer recall period (12 month) is likely to cause recall bias regarding injuries, especially if the injuries are less severe [20,26]. The two other questions selected for the latest version of the instrument measured the impact of the symptoms on school and/or work attendance and on sporting and/or recreational activity participation. These last two questions were selected to estimate the severity of the various reported conditions. The final version of the Teen Nordic Musculoskeletal Screening Questionnaire (TNMQ-S) developed for this study is presented as an Additional file 1.

### Validation of the questionnaire

Psychometric qualities of an instrument can be verified by measuring the : (1) validity, i.e. the capacity of the instrument to measure what it is suppose to measure; (2) reliability, i.e. the capacity of the instrument to produce constant results in two equivalent contexts; and (3) responsiveness, i.e. the capacity of the instrument to detect change [25]. This study assessed the criterion validity as well as the reliability of the TNMQ-S. Given the nature of the instrument (assessment of the 6-month prevalence of musculoskeletal symptoms and their impact), evaluating the responsiveness was deemed irrelevant for the present study.

### Test-retest reliability

Test-retest reliability can be measured by completing the same questionnaire twice in a time interval during which the participants’ condition remains unchanged [27]. Time interval for the present study was set at 24 to 48 hours to reduce the probability of new symptoms occurring between tests, and to facilitate participants’ compliance. In fact, if the time interval is too long, it is more likely that the individuals develop new symptoms between tests [28]. However, a shorter time interval can also be problematic, because participants might remember their answers to the first questionnaire and be inclined to duplicate them [28].

**Figure 1:**
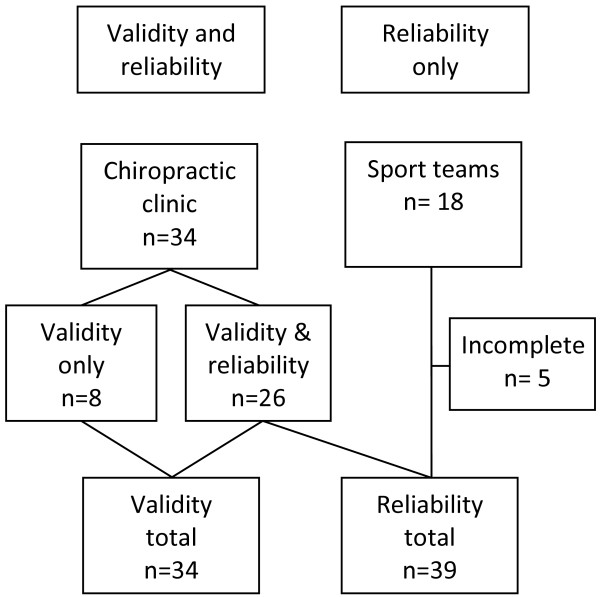
Participants recruitment flow chart.

Thirty-nine adolescents, with a mean age of 13.7 ± 1.8 years (see Table 1), were recruited from a University’s chiropractic outpatient clinic and local sport teams (Figure 1). They completed the questionnaire twice at an interval of 28.1 ± 8.0 hours. Before completing the questionnaires, all participants and their parents were informed of the procedures and gave their written informed consent. The study was approved by the Université du Québec à Trois-Rivières human research ethics committee and holds the certification number CER-11-174-06.03. The participants completed a first copy of the questionnaire in the waiting room before their appointment. Following the appointment, participants were sent home with an additional copy of the same questionnaire. They were instructed to complete the second copy of the questionnaire in the next 24 to 48 hours and return it at their next appointment. To increase sample size and diversity for the reliability phases of validation, two adolescent sport teams were also sought out to complete the questionnaires.

**Table 1:** Descriptive sample data

	**Test-Retest participants**	**Criterion validity participants**	**Total**
Sample (n)	39	34	48
Age (years)	13.97 ± 1.81	14.18 ± 2.25	13.94 ± 2.06
Gender			
Female (n)	21 (53.8%)	21 (61.8%)	27 (56.3%)
Male (n)	18 (46.2%)	13 (38.2%)	21 (43.8%)
Prevalence of symptoms, 6-month			
Neck	22 (57.9%)	25 (73.5%)	27 (57.4%)
Shoulders	12 (30.8%)	11 (32.4%)	13 (27.1%)
Upper back	17 (43.6%)	19 (55.9%)	21 (43.8%)
Elbows	2 (5.1%)	2 (5.9%)	2 (4.2%)
Wrists/Hands	10 (26.3%)	10 (30.3%)	10 (21.3%)
Low back	16 (42.1%)	20 (58.8%)	23 (48.9%)
Hips/thighs	9 (23.1%)	6 (18.2%)	10 (21.3%)
Knees	12 (29.3%)	12 (35.3%)	15 (31.3%)
Ankles/feet	17 (44.7%)	15 (45.5%)	20 (42.6%)

Answers to the matching questionnaires were compared and proportions of observed agreement (Po) were measured. Additionally, McNemar’s test was computed to assess the difference, or lack thereof, between paired proportions. McNemar’s statistical test is used to assess changes in peoples’ answers to dichotomous variables and detect if the changes tend to be positive (increased) or negative (decreased) [29]. The Cohen Kappa’s coefficient was calculated as an additional measure of agreement between tests. This statistic is further described in the criterion validity section.

### Criterion validity

Criterion validity is defined as the relationship between the results of the questionnaire and a point of reference considered a gold standard [25]. Thirty-three adolescents, mean age of 14.2 ± 2.3 years (see Table 1), completed a demographic questionnaire as well as the Teen Nordic Musculoskeletal Screening Questionnaire (Figure 1). Since the University’s chiropractic outpatient clinic’s clinical records are standardised and regularly audited, they were chosen as the point of reference to validate the questionnaire. The criterion validity was therefore measured by comparing the participants’ answers to the 6-month prevalence of musculoskeletal symptom question to their matching clinical records. The instrument is also meant to measure the severity of the symptoms by assessing their impact on school attendance and physical activity. Consulting a health professional is also an indicator of symptom severity [13]. Therefore, as an additional validity measure, the answers to the severity questions associated with a diagnosis found in the clinical record were compared to the answers not associated with a diagnosis which in turn should have been less severe. To simplify the analyses, the information found in the records was divided into three categories: 1) diagnosis, 2) reasons for consultation and past injuries or pain (not associated with a diagnosis) over the last 6 months and 3) symptoms mentioned in the questionnaire that were not associated with the two previous categories.

An independent clinician, not involved in the validation process and blinded to the study’s objectives, identified the most likely symptomatic regions associated with the diagnosis. These regions were then compared to their matching questionnaire section, and proportions (Po) of observed agreement were calculated. Since the participants sometimes had more than one diagnosis (multiple body regions affected), it was possible to obtain either a complete or a partial concordance between the diagnoses and the questionnaires. A complete concordance was achieved only if all the symptomatic regions of one participant associated with the diagnosis were also identified in the matching questionnaire. A partial concordance was obtained if at least one anatomical region associated with the participant’s diagnosis was identified in the matching questionnaire. The proportions of complete and partial concordance between the questionnaires and the reason for consultation and past symptoms reported in the clinical records were also measured. Because the agreement between observers (clinicians and participants) due to chance alone is impossible to detect if only the Po are used [30], the Kappa’s statistic was calculated. As explained by *Viera et al*. [30], the Kappa’s statistic qualifies the degree of agreement between observers. Whereas a Po and a Kappa of 1 both stand for a perfect agreement, a Po of 0.3 could be partially and totally ascribable to chance and a Kappa of 0.3 represents true (non-random) agreement to a degree of 30% over that due to chance. According to the same authors, a Kappa greater than 0.81 represents an almost perfect agreement; values ranging between 0.61 and 0.80 correspond to a substantial agreement, values between 0.41 and 0.60 are considered moderate and values lower than 0.40 are fair to poor. This statistic, however, might not be reliable for observations that do not occur often [30]. Therefore, the proportion of observed agreement should also be considered in order to correctly interpret the statistical results.

Finally, the difference between severe and less severe symptoms identified in the questionnaire that relate to: (1) the diagnosis or (2) the symptoms found in the questionnaire that cannot be associated with the information found in the clinical records, was measured using the Fisher’s exact test.

## Results

### Face validity

The layout of the original NMQ-E questionnaire, containing 99 questions on one page, seemed to be less than optimal to most adolescents. Indeed, several adolescents needed assistance to understand how to correctly answer the questionnaire, and in the end many questions remained unanswered. Thirty-six percent of the adolescents either answered incoherently or did not answer all of the questions when completing the translated NMQ-E in its original version. Furthermore, the life prevalence question was particularly difficult to answer because adolescents had poor recall of symptoms that occurred at a younger age. Thus, after considering the focus groups feedback and the face validity results, the questionnaire developed for this study was simplified and only three questions for each body region were selected for the final version: 1) a 6-month prevalence of symptoms question, 2) the impact of the symptoms on school and/or work attendance, and 3) the impact of symptoms on leisure and/or physical activity and sports.

### Test-retest reliability

Of the 52 adolescents included in the study, 13 did not return the second copy or were absent the day of the second questionnaire administration (Figure 1). Overall, test-retest reliability was good, the mean proportion of observed agreement (Po) ranged between 0.92 for the 6-month symptoms prevalence question and 0.99 for the impact of symptoms on school and work attendance question (Table 2). When each question was analysed separately, two thirds of the questions had a Po ≥ 0.95, and the lowest Po was 0.82 for the question regarding the prevalence of symptoms affecting the wrist or hand. The Cohen Kappa (k) results displayed mostly “substantial” to “almost perfect” agreement beyond chance. The lowest kappa obtained (k = 0.57) was for the wrist and hand symptom prevalence question which represented a “moderate” agreement. Almost half of the questions (44%) reached a “perfect or almost perfect agreement” (k = 0.81-1.00) while another 44% reached a “substantial agreement” (k = 0.61-0.80) and one question was considered of moderate agreement (k = 0.41-0.60). Every participant indicated “No” to the impact of symptoms on school attendance question regarding the elbow and the knee on both testing occasion. As a result, the Kappa and the McNemar’s statistics could not be calculated for those questions. The McNemar’s statistic results were all non-significant at P > 0.05. Therefore, none of the answers differed significantly between tests. Reliability results are presented in Table 2.

**Table 2:** Test-Retest reliability results (n = 39)

	** Musculoskeletal symptoms - 6 months**				** Impact of symptoms on school and/or work**				** Impact of symptoms on activities**			
	**n (%)**	**P**_**o**_	**Kappa**	**McNemar (P < 0.05)**	**n (%)**	**P**_**o**_	**Kappa**	**McNemar (P < 0.05)**	**n (%)**	**P**_**o**_	**Kappa**	**McNemar (P < 0.05)**
Neck	22 (57.9%)	0.97	0.95	N/S	2 (5.1%)	1.00	1.00	N/S	5 (12.8%)	0.92	0.68	N/S
Shoulders	12 (30.8%)	0.87	0.72	N/S	1 (2.6%)	1.00	1.00	N/S	5 (12.8%)	0.92	0.68	N/S
Upper back	17 (43.6%)	0.97	0.95	N/S	1 (2.6%)	0.97	0.66	N/S	5 (12.8%)	0.97	0.89	N/S
Elbows	2 (5.1%)	0.97	0.79	N/S	0 (0%)	1.00	*	*	2 (5.1%)	0.97	0.66	N/S
Wrists/Hands	10 (26.3%)	0.82	0.57	N/S	2 (5.3%)	0.92	1.00	N/S	5 (13.2%)	1.00	1.00	N/S
Low back	16 (42.1%)	0.95	0.89	N/S	2 (5.1%)	1.00	1.00	N/S	8 (20.5%)	0.92	0.75	N/S
Hips/thighs	9 (23.1%)	0.92	0.78	N/S	1 (2.6%)	1.00	1.00	N/S	4 (10.3%)	0.97	0.84	N/S
Knees	12 (29.3%)	0.87	0.69	N/S	0 (0%)	1.00	*	*	3 (7.7%)	0.95	0.64	N/S
Ankles/feet	17 (44.7%)	0.89	0.78	N/S	2 (5.3%)	0.97	0.65	N/S	7 (18.4%)	0.97	0.91	N/S
Questionnaire												
Po	0.92				0.99				0.96			

*McNemar’s statistic and Cohen kappa statistic could not be calculated because none of the adolescent presented elbow or knee problems having caused school or work absence.

### Criterion validity

Agreement obtained between the information found in the participant’s clinical record and the questionnaires was substantial with a Po = 0.71 and a k = 0.76 for the diagnosis. Concordance between clinical records, reason for consultation, and past pain or symptoms also yielded substantial agreement results with a Po = 0.70 and a k = 0.76. Table 3 details the criterion validity results.

**Table 3:** Agreement between the participant’s clinical record and the matching questionnaire (n = 34)

	**P**_**o**_		**Kappa**
	**Complete concordance**	**Partial concordance**	
Diagnosis*	20/28 (71.4%)	24/28 (85.7%)	0.76
Reason for consultation and past injury – last 6 months	16/23 (69.6%)	20/23 (87.0%)	0.76

*Referring to the number of diagnosed problems in the clinical records.

**Figure 2:**
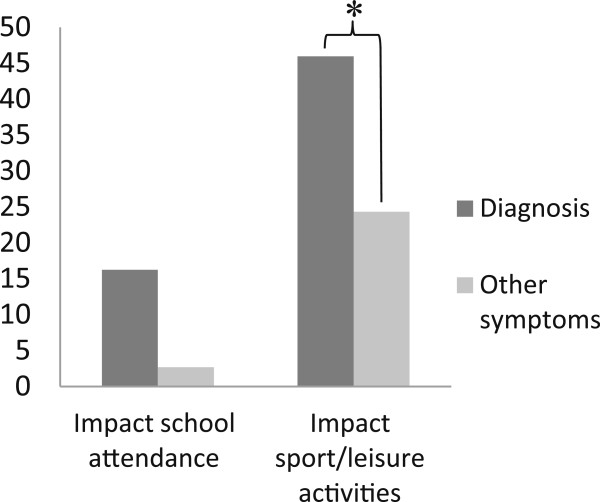
**Difference between the number of severe**^**1 **^**symptoms associated with a diagnosis or not. **^1^Severe symptoms refer to symptoms that caused physical activity reduction or school absence.

Figure 2 presents the difference between symptoms associated with a diagnosis and symptoms not associated with a diagnosis regarding physical activity reduction and school absence. The Fisher’s exact test revealed that more adolescents (χ^2^_(1)_ = 3.945, p = 0.054) missed school because of symptoms associated with a diagnosis compared to symptoms not associated with a diagnosis. Also, a significantly (χ^2^_(1)_ = 3,795, p < 0.05) greater amount of adolescents had to decrease their leisure and/or physical activity due to symptoms associated with a diagnosis (see Figure 2).

## Discussion

The purpose of this study was to develop a musculoskeletal symptom screening tool for younger populations derived from the NMQ-E [23] and other NMQ French versions [13,24], and to assess the reliability and the validity of this instrument. The final version of the study’s questionnaire includes three questions for each of the 9 body regions: the 6-month prevalence of musculoskeletal symptoms, the impact of these symptoms on school/work attendance as well as their impact on sport/leisure activities. The recall period chosen for the final version was 6 months because, as discussed earlier, a 12-month recall period is often affected by memory decay, and minor injuries are more likely to be forgotten [20,21].

The test-retest results with the 6-month recall period were encouraging and showed only slight fluctuations in responses. These fluctuations were presumably not due to changes in the participant’s health status, since the time interval between tests was short (28.1 ± 8.0 hours). It is however possible that the participants’ answers were slightly influenced by the clinician they consulted, since most participants completed one questionnaire before their consultation and the second one after their appointment. However, reliability results suggest that the questionnaire demonstrated a good overall stability of responses between tests with kappa values at moderate to perfect agreement beyond chance.

*Dawson et al*. assessed the reliability of the NMQ-E in an occupational cohort of 59 nursing students at a 24 hour interval [23]. This study had similar values for Po and slightly lower kappa reliability results for their 12-month prevalence question with a Po = 0.83-1.00 and k ≥ 0.55. These differences could be attributed to the recall period being longer (12 months) than the one used in the present study (6 months).

The TNMQ-S obtained reliable results regarding musculoskeletal symptoms described as ache, pain or discomfort. These results are not surprising, since adolescent self-reported past pain or injury has been shown to be reliable. *Grimmer et al. 2000*, found a very strong positive relationship between adolescents and their parents when reporting injuries occurring one week earlier [17]. Likewise, *Sundblad et al. 2006* reported high child–parent agreement results over a recall period of 7 to 11 weeks, either when adolescents were in absence of pain or injuries, when pain or injuries were severe or when the adolescents’ complaints were frequent. However, according to the same authors, minor injuries or pain and less frequent complaints were under-reported by the adolescents’ parents [18]. Another study found that adolescents rarely seek medical attention for their pain and injuries [31], which means that medical records are likely to underestimate the prevalence of minor pain or injury in the adolescent population. These findings suggest that questioning adolescents to obtain past pain and injury data would be more accurate for detecting minor pain or injuries or less frequent complaints than parental reports or medical records.

In the present study, concomitant validity as a measure of the criterion validity was assessed by comparing the 6-month symptoms prevalence question to the participants’ clinical record. Most diagnosed problems were detected by the questionnaire, with observed agreement of Po = 0.71 for the complete concordance (i.e. including all diagnosed symptoms) and Po = 0.86 for the partial concordance. Kappa values obtained for the criterion validity indicated substantial agreement beyond chance, indicating that there was good agreement between questionnaire items and clinical records [30]. However, some symptoms found in the questionnaire could not be linked to the clinical records. It is possible that the symptoms found in the questionnaires and not linked to the clinical records were minor problems undeclared to the clinician. In fact, when comparing diagnosed symptoms to symptoms not found in the clinical record, significantly more (P < 0.05) adolescents had reduced their physical or leisure activities due to symptoms associated with a diagnosis. Thus, it seems that adolescents only seek medical attention for pain and symptoms severe enough to have an impact on their physical activity. Indeed, *Watson et al*. [31] assessed the prevalence of back pain in schoolchildren of the United Kingdom, and found that adolescents were more likely to report pain of a greater intensity. The Teen Nordic Musculoskeletal Screening Questionnaire (TNMQ-S) was therefore useful to detect minor symptoms that adolescents did not necessarily report to their clinicians.

Concurrent validity for the impact of symptoms on school and physical activity questions could not be measured due to the lack of concordant information in the clinical records. Only a few clinical records had clear recommendations for sports and activity restrictions. Similarly, information regarding the number of school days lost could not be found in the clinical records.

### Study limitations

As mentioned earlier, criterion validation is measured by comparing the responses to questionnaires to a recognised gold standard. Since no recognised gold standard questionnaire measuring musculoskeletal symptoms in the adolescent population was found, the University’s outpatient clinic’s clinical records were used as the comparison for the criterion validation. Even though these records are standardised, regularly audited and provide an adequate level of details regarding patient musculoskeletal symptoms, they cannot be considered as a gold standard. Future studies should assess the construct validity rather than the criteria validity when no gold standard questionnaire is available.

## Conclusions

In summary, the Teen Nordic Musculoskeletal Screening Questionnaire (TNMQ-S) seems to be an appropriate tool to be used in the adolescent population as a self-administered musculoskeletal disorder screening tool. The study’s findings suggest that this instrument reports reliable results when self-administered to adolescents of 10 to 19 years of age. However, more validity testing should be undertaken regarding the impact of symptoms items. The TNMQ-S can be used to rapidly assess the presence of musculoskeletal symptoms and if these symptoms were severe enough to have caused school absence or physical activity reduction. This tool could serve as a musculoskeletal screening tool in certain descriptive epidemiological studies or it could also be integrated in future pain and symptom surveillance programs for schools or for individual and team sports.

### Consent

Written informed consent was obtained from the patients’ parents for the publication of this report and any accompanying images.

## Competing interests

The authors declare that they have no competing interests.

## Authors’ contributions

EPL participated in the study design, data analysis, experimentation and manuscript writing. MD participated in study design, manuscript writing and revision. VC participated in study design, manuscript writing and revision. All authors read and approved the final manuscript.

## Pre-publication history

The pre-publication history for this paper can be accessed here:

http://www.biomedcentral.com/1471-2431/14/173/prepub

## Supplementary Material

Additional file 1 **Teen Nordic Musculoskeletal Screening Questionnaire (TNMQ-S).** The additional file 1 is the TNMQ-S questionnaire presented in its validated and final form. Click here for file
