# Supplementary material for: Assessment of musculoskeletal symptoms and their impacts in the adolescent population: adaptation and validation of a questionnaire
Source: BMC Pediatr. 2014 Jul 3;14:173. doi: 10.1186/1471-2431-14-173 (PMC4107588; doi:10.1186/1471-2431-14-173)
Supplement: Additional file 1 — Teen Nordic Musculoskeletal Screening Questionnaire (TNMQ-S). The additional file 1 is the TNMQ-S questionnaire presented in its validated and final form. [file 1471-2431-14-173-S1.pdf]

Questionnaire de dépistage des symptômes musculo-squelettiques chez les adolescents

Teen Nordic Musculoskeletal Screening Questionnaire (TNMQ-S)

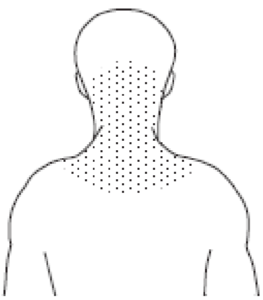

COU – NUQUE

Répondez aux questions suivantes en considérant la région ombragée sur cette image

1. Avez-vous ressenti des symptômes (douleur, élancement, gêne, inconfort) au **COU** et/ou à la **NUQUE** au courant des **6 derniers mois** ?
   Oui ☐ Non ☐ **si non, passez à ÉPAULES**

2. Au courant des **6 derniers mois :** avez-vous manqué une/des journées d'école ou de travail en raison de ce problème?
   Oui ☐ Non ☐

3. Au courant des **6 derniers mois :** avez-vous été contraint à réduire vos activités (sports et loisirs, etc.) en raison de ce problème?
   Oui ☐ Non ☐

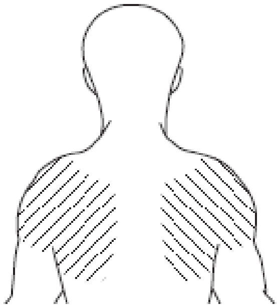

ÉPAULES

Répondez aux questions suivantes en considérant la région ombragée sur cette image

4. Avez-vous ressenti des symptômes (douleur, élancement, gêne, inconfort) aux **ÉPAULES** au courant des **6 derniers mois** ?
   Oui ☐ Non ☐ **si non, passez à HAUT DU DOS**

5. Au courant des **6 derniers mois :** avez-vous manqué une/des journées d'école ou de travail en raison de ce problème?
   Oui ☐ Non ☐

6. Au courant des **6 derniers mois :** avez-vous été contraint à réduire vos activités (sports et loisirs, etc.) en raison de ce problème?
   Oui ☐ Non ☐

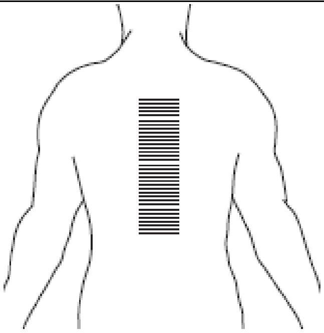

HAUT DU DOS

Répondez aux questions suivantes en considérant la région ombragée sur cette image

7. Avez-vous ressenti des symptômes (douleur, élancement, gêne, inconfort) au **HAUT DU DOS** au courant des **6 derniers mois** ?
   Oui ☐ Non ☐ **si non, passez à COUDES**

8. Au courant des **6 derniers mois :** avez-vous manqué une/des journées d'école ou de travail en raison de ce problème?
   Oui ☐ Non ☐

9. Au courant des **6 derniers mois :** avez-vous été contraint à réduire vos activités (sports et loisirs, etc.) en raison de ce problème?
   Oui ☐ Non ☐

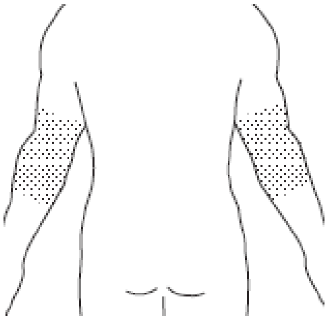

COUDES

Répondez aux questions suivantes en considérant la région ombragée sur cette image

10. Avez-vous ressenti des symptômes (douleur, élancement, gêne, inconfort) aux **COUDES** au courant des **6 derniers mois** ?
    Oui ☐ Non ☐ **si non, passez à POIGNETS-MAINS (prochaine page)**

11. Au courant des **6 derniers mois :** avez-vous manqué une/des journées d'école ou de travail en raison de ce problème?
    Oui ☐ Non ☐

12. Au courant des **6 derniers mois :** avez-vous été contraint à réduire vos activités (sports et loisirs, etc.) en raison de ce problème?
    Oui ☐ Non ☐

POIGNETS - MAINS

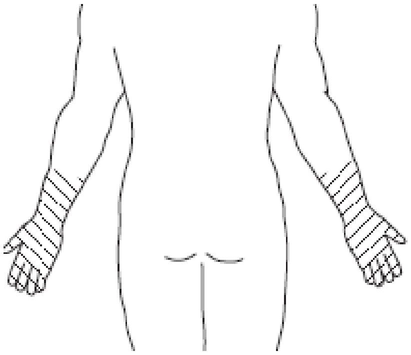

Répondez aux questions suivantes en considérant la région ombragée sur cette image

13. Avez-vous ressenti des symptômes (douleur, élancement, gêne, inconfort) aux **POIGNETS** et/ou aux **MAINS** au courant des **6 derniers mois** ?
Oui ☐ Non ☐ **si non, passez à BAS DU DOS**

14. Au courant des **6 derniers mois :** avez-vous manqué une/des journées d'école ou de travail en raison de ce problème?
Oui ☐ Non ☐

15. Au courant des **6 derniers mois :** avez-vous été contraint à réduire vos activités (sports et loisirs, etc.) en raison de ce problème?
Oui ☐ Non ☐

BAS DU DOS

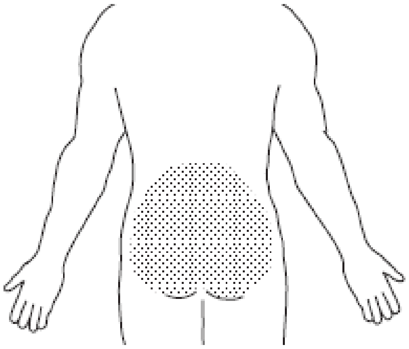

Répondez aux questions suivantes en considérant la région ombragée sur cette image

16. Avez-vous ressenti des symptômes (douleur, élancement, gêne, inconfort) au **BAS DU DOS** au courant des **6 derniers mois** ?
Oui ☐ Non ☐ **si non, passez à HANCHES - CUISSES**

17. Au courant des **6 derniers mois :** avez-vous manqué une/des journées d'école ou de travail en raison de ce problème?
Oui ☐ Non ☐

18. Au courant des **6 derniers mois :** avez-vous été contraint à réduire vos activités (sports et loisirs, etc.) en raison de ce problème?
Oui ☐ Non ☐

HANCHES – CUISSES

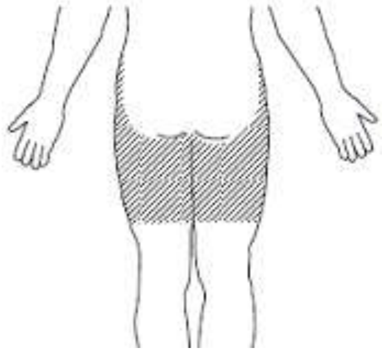

Répondez aux questions suivantes en considérant la région ombragée sur cette image

19. Avez-vous ressenti des symptômes (douleur, élancement, gêne, inconfort) aux **HANCHES** et/ou aux **CUISSES** au courant des **6 derniers mois** ?
Oui ☐ Non ☐ **si non, passez à GENOUX**

20. Au courant des **6 derniers mois :** avez-vous manqué une/des journées d'école ou de travail en raison de ce problème?
Oui ☐ Non ☐

21. Au courant des **6 derniers mois :** avez-vous été contraint à réduire vos activités (sports et loisirs, etc.) en raison de ce problème?
Oui ☐ Non ☐

GENOUX

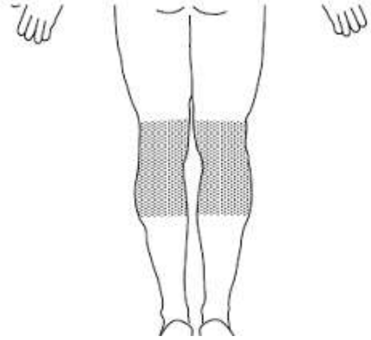

Répondez aux questions suivantes en considérant la région ombragée sur cette image

22. Avez-vous ressenti des symptômes (douleur, élancement, gêne, inconfort) aux **GENOUX** au courant des **6 derniers mois** ?
Oui ☐ Non ☐ **si non, passez à CHEVILLES-PIEDS (prochaine page)**

23. Au courant des **6 derniers mois :** avez-vous manqué une/des journées d'école ou de travail en raison de ce problème?
Oui ☐ Non ☐

24. Au courant des **6 derniers mois :** avez-vous été contraint à réduire vos activités (sports et loisirs, etc.) en raison de ce problème?
Oui ☐ Non ☐

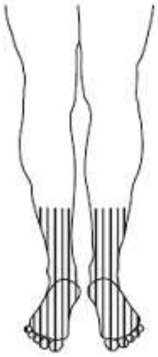

CHEVILLES - PIEDS

Répondez aux questions suivantes en considérant la région ombragée sur cette image

25. Avez-vous ressenti des symptômes (douleur, élancement, gêne, inconfort) aux **CHEVILLES** et/ou aux **PIEDS** au courant des **6 derniers mois** ?
   Oui ☐ Non ☐ **si non, vous avez terminé**

26. Au courant des **6 derniers mois :** avez-vous manqué une/des journées d'école ou de travail en raison de ce problème?
   Oui ☐ Non ☐

27. Au courant des **6 derniers mois :** avez-vous été contraint à réduire vos activités (sports et loisirs, etc.) en raison de ce problème?
   Oui ☐ Non ☐
